# De novo assembly of *Phlomis purpurea* after challenging with *Phytophthora cinnamomi*

**DOI:** 10.1186/s12864-017-4042-6

**Published:** 2017-09-06

**Authors:** Aladje Baldé, Dina Neves, Francisco J. García-Breijo, Maria Salomé Pais, Alfredo Cravador

**Affiliations:** 1 Plant Molecular Biology and Biotechnology Lab, Center for Biosystems (BioSys), Functional and Integrative Genomics (BioFIG), Edifício C2, Faculdade de Ciências da Universidade de Lisboa, Campo Grande, 1749-016 Lisbon, Portugal; 2 Present Address: Universidade Jean Piaget, Bissau, Guinea-Bissau; 3 Faculdade de Ciências e Tecnologia, Universidade do Algarve, Campus de Gambelas, 8005-139 Faro, Portugal; 4 Departamento de Ecosistemas Agroforestales, Universidad Politécnica de Valencia, Camino de Vera s/n, 46022 Valencia, Spain; 5 Centre for Mediterranean Bioresources and Food (MeditBio), FCT, Universidade do Algarve, Campus de Gambelas, 8005-139 Faro, Portugal

**Keywords:** *Phlomis purpurea*, Transcriptomics, *Phytophthora cinnamomi*, Resistance, Defence response, Time course challenge, Casparian strips, Cutin

## Abstract

**Background:**

*Phlomis* plants are a source of biological active substances with potential applications in the control of phytopathogens. *Phlomis purpurea* (Lamiaceae) is autochthonous of southern Iberian Peninsula and Morocco and was found to be resistant to *Phytophthora cinnamomi. Phlomis purpurea* has revealed antagonistic effect in the rhizosphere of *Quercus suber* and *Q. ilex* against *P. cinnamomi*. *Phlomis purpurea* roots produce bioactive compounds exhibiting antitumor and anti-*Phytophthora* activities with potential to protect susceptible plants. Although these important capacities of *P. purpurea* have been demonstrated, there is no transcriptomic or genomic information available in public databases that could bring insights on the genes underlying this anti-oomycete activity.

**Results:**

Using Illumina technology we obtained a de novo assembly of *P. purpurea* transcriptome and differential transcript abundance to identify putative defence related genes in challenged versus non-challenged plants. A total of 1,272,600,000 reads from 18 cDNA libraries were merged and assembled into 215,739 transcript contigs*.* BLASTX alignment to Nr NCBI database identified 124,386 unique annotated transcripts (57.7%) with significant hits. Functional annotation identified 83,550 out of 124,386 unique transcripts, which were mapped to 141 pathways.

39% of unigenes were assigned GO terms. Their functions cover biological processes, cellular component and molecular functions. Genes associated with response to stimuli, cellular and primary metabolic processes, catalytic and transporter functions were among those identified.

Differential transcript abundance analysis using DESeq revealed significant differences among libraries depending on post-challenge times.

Comparative cyto-histological studies of *P. purpurea* roots challenged with *P. cinnamomi* zoospores and controls revealed specific morphological features (exodermal strips and epi-cuticular layer), that may provide a constitutive efficient barrier against pathogen penetration. Genes involved in cutin biosynthesis and in exodermal Casparian strips formation were up-regulated.

**Conclusions:**

The de novo assembly of transcriptome using short reads for a non-model plant, *P. purpurea*, revealed many unique transcripts useful for further gene expression, biological function, genomics and functional genomics studies.

The data presented suggest a combination of a constitutive resistance and an increased transcriptional response from *P. purpurea* when challenged with the pathogen*.* This knowledge opens new perspectives for the understanding of defence responses underlying pathogenic oomycete/plant interaction upon challenge with *P. cinnamomi*.

**Electronic supplementary material:**

The online version of this article (doi:10.1186/s12864-017-4042-6) contains supplementary material, which is available to authorized users.

## Background

The genus *Phlomis* (Lamiaceae), which includes over 100 species, is native to the Mediterranean region and from central Asia to China. Some species are cultivated as ornamentals and many of them are used in traditional medicine [1]. *Phlomis purpurea* Linnaeus (purple phlomis), in particular, is indigenous to the southern Iberian Peninsula and Morocco and grows in habitats infested by *Phytophthora cinnamomi. Phytophthora cinnamomi* is a highly aggressive oomycete that causes root rot disease in thousands of plant species including ornamentals and crop plants as well as fruit and forest trees [2]. This pathogen is the main biotic factor responsible for *Quercus suber* and *Q. ilex* subsp. *rotundifolia* decline in the southwest region of Iberian Peninsula [3] constituting a severe threat to the cork and holm oak agro-ecosystems. A survey of the *P. cinnamomi* infested habitats in Algarve, including cork oak stands, led to the discovery of some plant species not infected by this oomycete [4]. Among them *Phlomis purpurea* proved to be not only host for *P. cinnamomi* [5] but also and noticeably to inhibit the pathogen hyphae to penetrate beyond the surface layer of the root epidermis [6]. Moreover, *P. purpurea* root extracts reduce the production of *P. cinnamomi* disease cycle structures and prevent germination of chlamydospores and zoospores, suggesting the ability of *P. purpurea* to reduce *Q. suber* root infection by *P. cinnamomi*, thus having the potential to reduce disease spread [5]. *Phlomis purpurea* plants grown in glasshouses reduce growth of the soil pathogen inoculum. A significant reduction of the root rot disease symptoms in *Q. suber* has been observed when *P. purpurea* is growing in the tree surroundings which accounts for the importance of the triangular interactions between *Phlomis purpurea, Phytophthora cinnamomi* and *Q. suber/Q. ilex* previously reported by Neves et al. [5] and Neves [6]. A novel triterpenoid (phlomispurpentaolone), recently isolated from *P. purpurea* roots and exuded to the rhyzosphere, exhibits anti-*Phytophthora* and antitumor activities [7]. More than 150 different compounds including sesquiterpenoids, diterpenoids, triterpenoids, triterpene saponins and flavonoids have been identified in the genus *Phlomis* [1, 8]. Some of them are structurally related to phlomispurpentaolone and exhibit activity against cancer cells which suggests that they have properties similar to those described for the novel triterpenoid identified by Mateus et al. [7]. Plant saponins may function as phytoantecipins, having constitutive or pre-infection defence properties, or as phytoalexins, ensuring post-infection defences against pathogens [9]. *Phlomis purpurea* was used as a household detergent in the past, suggesting that this plant is rich in saponins. In addition to chemical strategies, preformed physical barriers may also account for the apparent type I nonhost resistance exhibited by *P. purpurea* to *P. cinnamomi*. Casparian strips are cell-wall modifications hypothesized to be crucial for selective nutrient uptake and also exclusion of pathogens [10]. They were first described in 1865 by Caspary [11] but only recently the biosynthetic mechanisms that govern their localized deposition have been addressed [12]. Lignin, the main component of Casparian strips [13, 14], is known to be associated with plant resistance to pathogens [15, 16]. The pathways involved in defence of *Phlomis* plants against fungi are unknown. Characterizing changes in gene expression triggered by the pathogen in the resistant *P. purpurea* plant may contribute to elucidate the mechanisms underlying constitutive defences of *P. purpurea* against *P. cinnamomi* infection.

In the present paper we make use of the millions of generated paired-end Solexa reads that reveal the transcriptomic profile of *P. purpurea* challenged with *P. cinnamomi* along a time course experiment. Differential gene expression profiles were compared using cDNA libraries from *P. purpurea* plants at six time points after challenging with *P. cinnamomi*. Quantitative real time PCR (qRT-PCR) was further applied to ten differential expressed candidate genes to evaluate the robustness of the sequencing-based approach. The morphological data on *P. purpurea* challenged with *P. cinnamomi* and control have been jointly discussed with the molecular data. An abstract of the results here reported has previously been presented [17].

## Results

### Histology

**Fig. 1:**
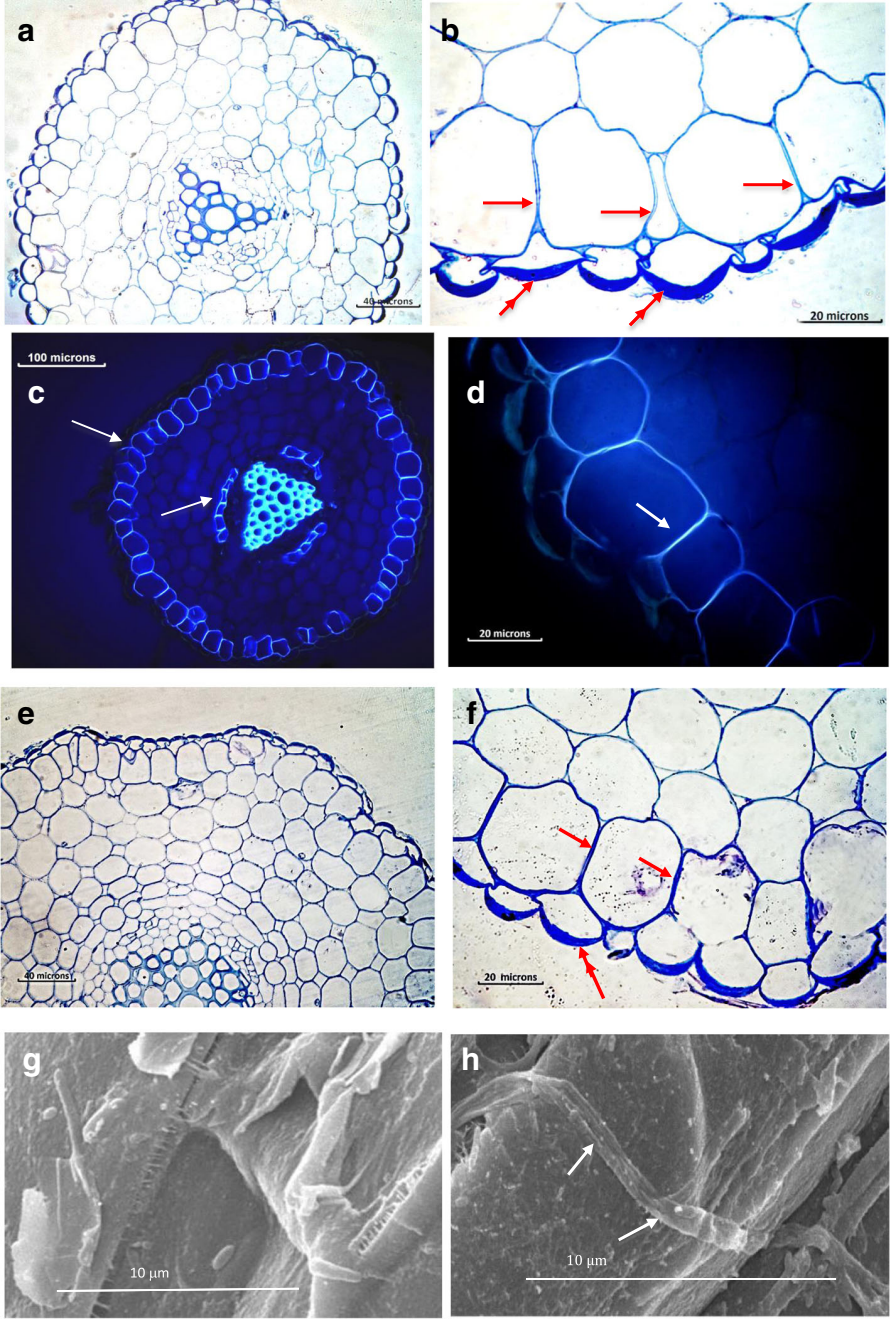
Histology of control and challenged roots of *Phlomis purpurea.* **a** and **b**: Cross section of a control root of *Phlomis purpurea* stained with toluidine blue showing the presence of lignin evidenced by the bright blue colour (**a**) and the reinforcement (double arrows) over the outer tangential wall of the epidermal cells (**b**). An exodermis whose radial walls have Casparian strips is evident in **b** (arrows). **c** and **d**: Paraffin embedded sections of control roots of *P. purpurea*, stained with direct red and observed under UV light. An intense blue light fluorescence evidence the Casparian strips in the exodermis and endodermis (arrows). In **d** is shown a magnification of Casparian strips (arrow). **e** and **f**: Cross section of a *P. purpurea* root challenged with *Phytophthora cinnamomi* along 72 h. Although no differences were observed in radial walls of the exodermis between control and challenged plant roots, a statistically significant reinforcement of the outer tangential walls was detected in challenged ones (compare with **a** and **b** and see Additional file 1). **g** and **h:** Low Temperature Scanning Electron Microscopy (LTSEM) of *P. purpurea* root epidermis. In **g** view of the outer tangential wall of the epidermal cells of *P. purpurea* root; in **h** *P. cinnamomi* hyphae growing over the outer tangential wall (arrows)

*Phlomis purpurea* control root sections stained with toluidine blue show a high thickening of the outer tangential wall of the epidermal cells (Fig. 1a and b). Casparian strips are clearly observed in the exodermis and endodermis of control root cells stained with direct red and observed under UV light (Fig. 1c and d). Statistical analysis of measurements taken on 50 samples showed no significant differences (*P =* 0.095) in the thickness of the radial walls of the exodermis and a significant (*P <* 0.0001) increase in the thickness of the external tangential root cells walls of plants challenged with *Phytophthora cinnamomi* (Fig. 1e and f). Scanning micrographs reveal the hyphae of *P. cinnamomi* growing over the epidermal cells without penetrating this potent physical barrier (Fig. 1g and h).

### Transcriptomics

#### *Paired-end sequencing and* de novo *assembly*

A total of 1,272,600,000 reads were obtained from 18 cDNA libraries (constructed from the 18 samples) of mRNA whole transcriptome sequencing of *P. purpurea*. The average length of the reads was 172 nucleotides. The length of the unigenes generated from the de novo assembly ranged from 155 to 7762 bp (Table 1) and the mean sequence length was 756. The N50 value was 620 bp. The frequency distribution of lengths is shown in Additional file 2: Figure S1. For gene expression analysis only unigenes with size above 200 bp were used.

**Table 1:** Length distribution of *Phlomis purpurea* unigenes generated by the de novo assembly

Length distribution of transcript contigs	
Mean sequence length:	756.25 ± 723.99 bp
Minimum length:	155 bp
Maximum length:	7762 bp

Results generated with a Kmer of 39 were taken based on the quality control parameter N50 and the number of unused reads. After removing the assembled transcripts shorter than 200 nt we generated 215,739 assembled transcripts, with lengths ranging from 200 to 7762. A high proportion of unigenes (43%, corresponding to 92,640 transcripts) had a length between 200 and 500 bp. The length distribution and number of *P. purpurea* unigenes longer than 200 bp are shown in Table 2.

**Table 2:** Length distribution and number of *Phlomis purpurea* unigenes ≥200 bp

Length of unigenes (bp)	Number of unigenes
200–500	92,640
500–1000	78,549
1000–1500	31,684
1500–2000	7162
≥ 2000	5704

The length of most of the annotated unigenes (58.3%) ranged between 200 and 4000 bp.

#### Annotation

Unigenes were submitted to BLASTX similarity searches to assess the putative identities of the assembly. A similarity search against the NCBI database [18] using an *E-*value threshold of 10^−5^ showed that out of the 215,739 contigs, 124,386 (57.7%) exhibited significant similarity with proteins in the Nr (non-redundant) database. In order to identify the biological pathways active in *P. purpurea* genes, the de novo assembly was mapped against the eukaryote sequence database at KEGG [19]. In total, 83,550 out of 124,386 transcripts presented significant similarity with gene ontology (GO) pathways.

The *E*-value distribution of the top hits in the Nr database revealed that 66.9% of unigenes with *E-*value *≤* e^−50^ were longer than 500 bp. The model plants from which most of the unigenes (48,711 out of 83,550) retrieved an annotation are listed in Additional file 3: Figure S2.

GO annotations for 83,550 unigenes revealed terms from “Biological processes” (27,053 unigenes), “Cellular component” (23,541 unigenes) and “Molecular function” (32,956 unigenes) (Additional file 4: Figure S3). 132,189 out of 215,739 unigenes (61.3%) did not show any significant hit with established GO terms, largely due to their uninformative (e.g. unknown, putative, or hypothetical) protein description. Within the “Biological process” category, the most represented functions correspond to “Oxidation-reduction processes”, “Translation” and “Metabolic processes” (around 6000, 2900 and 2900 contigs, respectively). Most unigenes of the “Cellular component” category return hits to genes matching functions at “Cell membrane” and “Nucleus components” levels (about 5250 and 4000 contigs, respectively). In the “Molecular function” category, genes related to “ATP binding” and “Nucleotide binding” functions (about 8640 and 4080 contigs, respectively) are predominant.

**Fig. 2:**
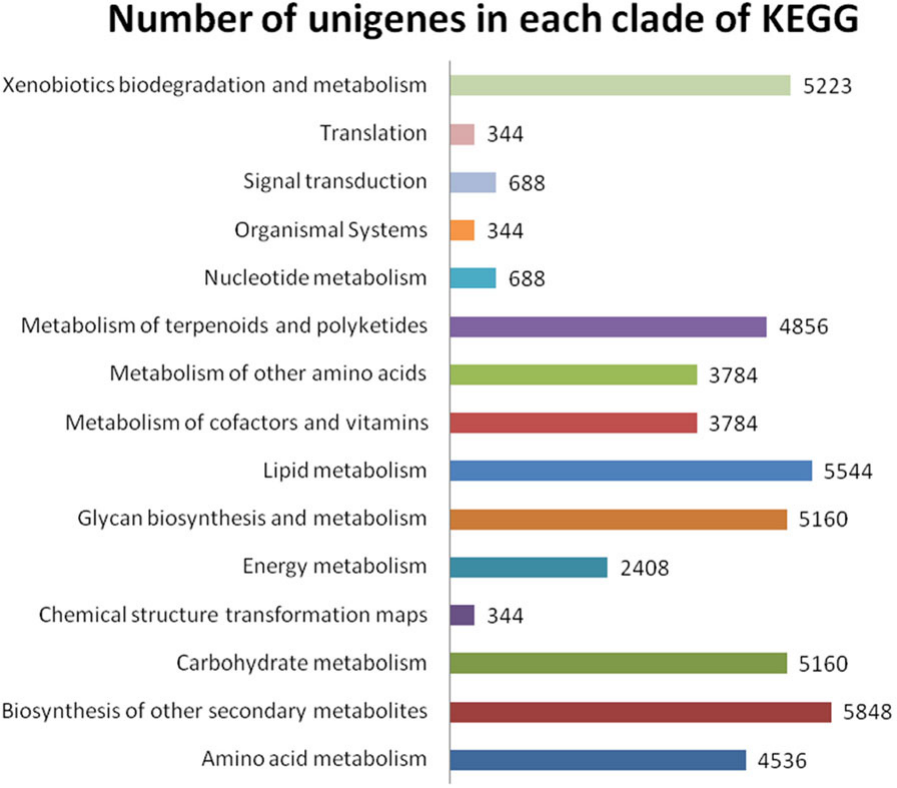
Number of unigenes in each clade of the KEGG pathway maps. The unigenes were assigned 141 KEGG pathways within 15 clades under major categories: Metabolism, Genetic information processing, Environmental information processing, Cellular processes and Organismal systems

The assembled unigenes were further annotated against the KEGG database [19] to obtain the Enzyme Commission number (EC). In total, 48,711 out of 83,550 (58.3%) annotated unigenes were assigned to 141 KEGG pathways. These pathways are grouped into fifteen clades under five major KEGG categories, namely Metabolism, Genetic information processing, Environmental information processing, Cellular processes and Organismal systems. Terpenoids and polyketides metabolism, biosynthesis of other secondary metabolites, lipid metabolism, glycan biosynthesis and metabolism, carbohydrate metabolism as well as xenobiotics metabolism and biodegradation were the top six pathways more represented by the unigenes annotated (Fig. 2).

#### GC content analysis of Phlomis Purpurea transcriptome

The GC content of the *P. purpurea* transcripts ranges from 26 to 73% with the mean being 43.18%. *Phlomis purpurea* transcripts have GC levels with a unimodal distribution typical of coding sequences in eudicots. However, GC level is centered (43%) below the usual range observed for eudicots (44–47%) [20] and the GC content range is broader when compared with other eudicots namely from Brassicaceae (*Arabidopsis* 40–60%; *Glycine max* 40–65%), Fabaceae (*Pisum sativum* 35–55%;) and Solanaceae (*Nicotiana tabacum* 35–55%; *Lycopersicon esculentum* 35–65%; *Solanum tuberosum* 25–60%) families. The distribution of the GC content and its frequency are shown in Additional files 5 and 6: Figure S4 and Table S1).

### Analysis of differentially expressed genes using DESeq

**Fig. 3:**
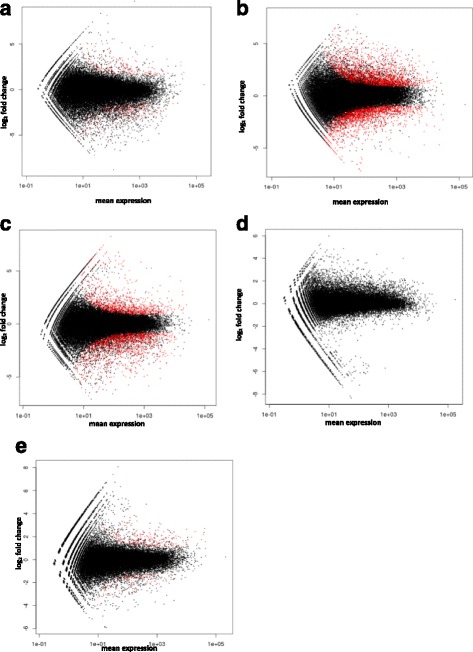
Differential expressed genes analysis. Plot of normalized mean versus log2 fold change for the contrast between *Phytophthora cinnamomi*-control versus *P. cinnamomi*-challenged *Phlomis purpurea*. Genes that are significant at 10% are colored in red. Red dots mark contigs detected as being significantly differentially expressed at a 10% false discovery rate with Benjamini–Hochberg multiple testing adjustments (*P* < 0.01). Normalized mean between zero and 6 h (**a**), 6 and 12 h (**b**), 12 and 24 h (**c**), 24 and 48 h (**d**), 48 and 72 h (**e**)

The differential expression patterns among libraries revealed that the highest difference in expression occurs between 6 and 24 h post challenge (Fig. 3). Between 24 h and 48 h post-challenge, differential expression was not significant (Fig. 3d) and between 48 h and 72 h a slight difference in expression occurred (Fig. 3e).

Additional files 7 to 13 list some genes up-regulated in the transcriptome of *Phlomis purpurea* at 12 and 24 h post-challenged with *Phytophthora cinnamomi*. Additional files 7 and 8: Tables S2 and S3) show the genes up regulated at 24 and 12 h post challenge (fold increase > 2, column E). Only 698 or 1702 of these genes are up-regulated at a level statistically significant at 24 and 12 h, respectively (Padj <0.05, from column H in the Additional files 9 and 10: Tables S4 and S5). Additional files 11 and 12 show the genes that are up-regulated with a fold increase > 4 at 24 and 12 h post challenge, respectively (column E in Tables S6 and S7). 410 or 500 genes were up-regulated with a fold increase > 10, at 24 and 12 h post challenge (Additional files 13 and 14: Tables S8 and S9, respectively).

Table 3 lists unigenes involved in metabolic pathways of cutin biosynthesis and Casparian strips formation, as well as in other active or induced defence responses and shows the increase and significance of their up-regulation at 12 and 24 h after challenge with *Phytophthora cinnamomi,* respectively.

**Table 3:** *Phlomis purpurea* genes putatively involved in structural barriers and induced defence responses against *Phytophthora cinnamomi*

Contig		Putative identity	Reported function or role, notes	fold change	padj^a^	fold change	padj^a^	Ref
12 h	24 h			12 h		24 h		
21722	113347	cytochromes P450 enzyme	lignin biosynthetic pathway, membrane protein	40.3	0.001	10.7	2.54 e-06	[43, 44]
66810	-	β-ketoacyl-CoA synthase/reductase	cutine biosynthesis	30.5	7.71 e-14	-	-	[46, 47]
61695	57161	1-acyl-sn-glycerol-3-phosphate/acyltransferase	cutine biosynthesis	2.4	0.04	2.5	0.006	[46, 47]
85447	27377	o-acyltransferase	BAHD acyltransferases superfamily biosynthesis cutine/suberine	57.8	1.97 e-05	2.2	0.025	[48]
5933	13339	hydroxycinnamoyltransferase	BAHD acyltransferases superfamily involved in cutin and suberin synthesis	2.5	0.01	2.9	0.7	[48]
5854	-	hydroxycinnamoyl-CoAacetyltransferas	BAHD acyltransferases superfamily (idem)	2.6	0.04	-	-	[48]
27011	83517	desacetoxyvindoline acetyltransferase	BAHD acyltransferases superfamily (idem)	6.9	0.27	2.7	0.07	[48]
9911	53108	myb	regulators of cuticle biosynthesis	3.2	0.001	12.1	0.0002	[49]
62249	52265	myb-related		4.7	0.02	2.3	0.3	[50]
66721	131269	myb-like		2.5	0.05	3.2	1	[50]
9580	76701	r2r3-myb	regulation of cell wall biosynthesis	6.2	0.1	2.09	0.7	[52]
5647	56253	abc (ATP-Binding Cassette)	transporter in cuticular lipid export	6.5	1.15 e-07	3.1	0.0002	[46, 55]
2081	-	xyloglucan:xyloglucosyl transferase	restructuring and integration in cell walls	7.3	2.42 e-10	-	-	[56]
64844	-	glycosyltransferase	catalyzes transfer of sugars from nucleotide DP to a nucleophilic glycosyl acceptor	7.7	0.0006	-	-	[56]
80942	87785	α-expansin	catalyze extension of cell walls	5.4	0.1	3.9	0.07	[57]
837	-	expansin-like		3.4	0.0002	-	-	[57]
7846	36167	cinnamoyl CoA:NADP oxidoreductase (CCR)	catalyzes 1st step synthesis of lignin monomers	11.9	0.0007	2.3	0.13	[60]
25358	-	cinnamyl alcohol dehydrogenase	biosynthetic route to monolignols	6.9	0.004	-	-	[61]
10016	-	cinnamate 4-hydroxylase	lignin biosynthetic pathway, membrane protein	7.3	5.34 e-10	-	-	[62]
1432	-	4-coumarate 3-hydroxylase	lignin biosynthetic pathway, membrane protein	6.5	1.17 e-07	-	-	[62]
1081		phenylalanine ammonia-lyase	biosynthetic route toward monolignols	7.1	0.00016			[62]
26215	8769	laccase	monolignol polymerization	11.6	0.0002	5.9	2.8 e-07	[63]
21125	-	lacase-14		13.6	0.17	-	-	
-	70446	laccase-like		-	-	6.4	0.05	
-	70447	laccase-13		-	-	2.7	1	
13754	-	laccases 17		3.2	0.35	-	-	[64]
781	7923	peroxidase	monolignol oxidation	3.6	0.0005	5.2	2.24 e-06	[12]
20241	99047	CASP-like	Scaffolding activities involved in subcellular precision of lignin polymerization	4.4	0.05	6.0	0.9	[66]
5106	14096	AP2	Transcription factor. Play various roles including response to biotic and environmental stress.	17.7	0.0009	3.1	0.01	[72, 73]
2245	53577	GDSL (esterases/lipases)	Involved in defence from pathogens and stress, plant development, morphogenesis	30.9	4.18 e-22	9.92	4.2 e-08	[74, 77]
51706	-	lipase		6.2	0.007	-	-	[76, 77]
766	9217	phospholipase		11.0	1.13 e-09	2.4	0.009	[76, 92]
-	24143	esterase		-	-	8.9	0.0004	[76, 77]
15968	-	cc-nbs-lrr	resistance-genes in plant response to pathogens	7.7	0.0006	-	-	[82, 83]
64844	-	glycosyltransferase	catalyzes transfer of sugars from nucleotide DP to a nucleophilic glycosyl acceptor	7.7	0.0006	-	-	[78]
12279	69649	udp-glycosyltransferase	catalyzes conversion of cycloartenol to steroidal saponins	11.1	1.01 e-14	3.5	0.0016	[79]
1718	-	farnesyl synthase	involved in biosynthetic route to triterpenes	2.7	0.001	-	-	[79]
13713	-	squalene synthase	involved in biosynthetic route to triterpenes	3.3	0.0001	-	-	[78, 79]
-	8585	cyclase	catalyzes cyclisation of oxidosqualene a step of the triterpene biosynthesis	-	-	2.8	0.002	[78, 79]
54620	-	brassinosteroid	acceptor of UGT enzymes that glycosylate triterpenes (saponin biosynthesis)	8.7	2.23 e-06	-	-	[79]
24145	-	β-amyrin synthase	part of plant triterpene biosynthetic cluster	2.2	0.02	-	-	[785,79]
62788	-	terpene	enzymes involved in terpene metabolism	23.6	7.31 e-15	-	-	[78, 79]
51457	-	pathogenesis-related proteins	acting as chitinases, glucosidases, peroxidases	6.2	0.0004	-	-	[84]

The fold change and the *p*-value (padj) are shown in separate columns for transcripts up-regulated at 12 and 24 h post-challenge. A dash indicates no contig was found corresponding to a given putative identity at a specified post-challenge time point

^a^Only the lowest *p*-value is shown whenever more than one contig were found to have the same assignment regardless the value of their fold change

### Quantitative real-time PCR analysis

**Fig. 4:**
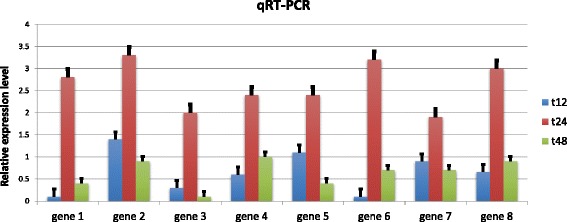
Relative expression of selected *Phlomis purpurea* transcripts by quantitative real-time PCR at three post-challenge times. The following selected genes are putatively related to *P. purpurea* response to the pathogen Gene1: wall-associated receptor-kinase 5-like; gene2: cinnamyl-alcohol dehydrogenase; gene3: hydroxyproline-rich glycoprotein; gene4: cyanidin-3-O-glucoside 2-O-glucoronosyltransferase-like; gene5: resistance protein rgc2; gene6: subtilisin-like protease; gene7: calcium-binding mitochondrial carrier protein aralar1-like; gene8: serine-threonine protein plant. Transcript quantitative expression was normalized with the reference genes β-actin, succinate dehydrogenase and elongation factor1α. Error bars correspond to the standard deviation of the mean of four technical replicates

To assess the reliability of our sequencing-based approach for identifying *Phytophthora*-responsive genes in *Phlomis purpurea*, the expression level of eight candidate genes was assessed by qRT-PCR. The expression profiles of these genes agree with the predictions from the Illumina sequencing results. The confirmed candidates were differentially expressed at least in one of the three time points. For the eight genes analysed, the highest variation revealed by qRT-PCR occurred at 24 h post-challenge (Fig. 4) which is in agreement with Illumina results.

## Discussion

Reference sequences are not available for many organisms. However, the de novo assembly of transcriptomes using short reads has received attention and transcriptome studies in non-model organisms have been conducted [21–24]. Several tools have been developed to allow de novo short read assembly such as Trinity [25], Velvet [26], Oases [27], Trans-ABySS [28], SOAPdenovo-Trans [29]. In this study, we report on a strategy for de novo assembly of a transcriptome using short reads for *Phlomis purpurea*, a non-model plant*.* An in-depth analysis of its transcriptome was performed using RNA-Seq technology, carrying out de novo transcriptome assembly with the Velvet/Oases package [26, 28].

The longest unigenes generated from the de novo assembly were 7762 bp. The number of short unigenes with length between 200 to 500 bp was the highest (92,640) corresponding to 43% of total unigenes (Table 2). The following reasons can explain this result: (1) the total RNA used for sequencing was pooled from three samples, which may have negatively affected the reads assembly [30]; (2) the high dynamic range of mRNA expression can be a problem for comprehensive de novo mRNA sequencing and assembly [30]; (3) the high frequencies of alternative splicing and fusion events may have restricted the assembly of short sequences into longer ones [31, 32].

A high average transcriptome coverage and depth was obtained for the reads generated from 18 libraries sequenced using paired-end Illumina HiSeq2000 sequencer, ensuring the reliability of the RNA-Seq for gene expression profiling. Cell type and developmental stage were kept exactly the same for all the samples used for RNA extraction. This is a pre- requisite for every RNA-Seq experiment as a way to reduce the risk of coverage variability across transcripts [33]. However, it is impossible to know the true coverage of the *P. purpurea* gene space since its complete genome sequence is not available.

In this study, a total of 48,711 annotated protein-coding genes were predicted using Blast2GO [34]. The analysis of sequence conservation helps in transferring knowledge from model plants to *P. purpurea* and in identifying candidate genes. A large number of *P. purpurea* transcripts showed significant similarity with *Vitis vinifera*. The 42.3% unigenes of *P. purpurea* transcriptome exhibit no significant similarity against the Nr NCBI database. It is quite common to find un-characterized sequences from cDNA libraries, especially in studies involving large-scale sequencing [21, 22, 30, 35]. Their proportion ranges more commonly between 35 and 50%. Studies on apple and strawberry EST sequences characterization revealed that approximately 40% [36] and 43.9% [37] of the unique transcripts from the respective assembly did not present significant similarity. The high percentage of the *P. purpurea* transcripts with no hits to the Nr protein database may be due to the high percentage of short unigenes and may constitute a potential for the discovery of new genes from *P. purpurea* and the identification of new gene networks.

At the best of our knowledge, the dataset obtained through de novo assembly is the first transcriptomic data of *Phlomis* genus generated from massively parallel sequencing.

### Outer tangential wall

The presence of a thick outer tangential wall over the epidermal cells of *Phlomis purpurea* control and of *P. purpurea* roots challenged *with Phytophthora cinnamomi,* suggests a constitutive morphological protection of the epidermal root cells. As shown in Fig. 1 and confirmed through numerous observations, *P. cinnamomi* hyphae are unable to penetrate this outer tangential wall of epidermal root cells. Moreover, the pathogen was never isolated from challenged roots. In spite of extensive lignification, resistant species have always been shown to present lesions produced by the oomycete growth before becoming restricted [38, 39].

According to Ashford et al. [40], the outer tangential epidermal cell wall presents an unusual structure, consisting of several distinct wall layers with some of them appearing spongy. There are several potential roles for the thickened wall of the epidermal cells such as providing a protective function, influence the water relations within the cell and/or provide a substrate for fungi to enable hyphal outgrowth [40]. The presence of thick tangential epidermal cell walls has also been reported for *Wollsia pungens* mycorrhizal hair roots [41]. The authors have exhaustively studied the composition of the external epidermal cell layer and demonstrated the arrangement of microfibrils typical of secondary cell walls, with high abundance of β-glucans and galactose side-chains [41]. The composition of the outer tangential epidermal cell walls of *Vaccinium corimbosum* fruits and the identification of a delimited transition between the cuticle, the cutinized layer and the cellulosic layer have also been reported [42].

### Candidate genes putatively involved in cutin and Casparian strips formation

The following discussion is essentially based on genes encoding proteins related to the biosynthesis of cutin (one of the main components of the plant cuticle) and components of Casparian strips that appear up-regulated at 12 and 24 h after challenging the roots of *Phlomis purpurea* with *Phytophthora cinnamomi* (Table 3)*.*

#### Cutin

Cutin and suberin are the polymer matrices for lipophilic cell wall barriers. These barriers control the fluxes of gases, water and solutes, and are involved in the protection of plants from biotic and abiotic stresses (for reviews see [43, 44]). Cutin breakdown products may constitute potential signals perceived by the plant as first elicitors, thus providing a way for the activation of the plant immune system [45]. Cutin, attached to the epidermal cell walls is composed of inter-esterified hydroxy and hydroxy-epoxy fatty acids. The availability of high throughput sequencing technologies was responsible for a tremendous increase in the identification of genes related to cutin biosynthesis that begins with de novo fatty acid synthesis. The identification of genes coding for cytochrome enzymes (CYPs), β-ketoacyl CoA synthase, *sn-*glycerol-3-phosphate acyltransferase (GPAT) accounts for their role in the first steps of the biosynthesis of cutin monomers as recently reported [46]. The genes coding for β-ketoacyl-CoA reductase and enoyl-CoA reductase are in the base of the formation of long chain fatty acids that are important components of the cuticle [47]. BAHD (*B*enzylalcohol acetyltransferase, *a*nthocyanin *O*-hydroxycinnamoyltransferase, anthranilate *N*-*H*ydroxycinnamoyl/benzoyltransferase, *D*eacetylvindoline acetyltransferase) acyltransferases superfamily genes are among those up-regulated after challenge of *P. purpurea* with *P. cinnamomi* (as suggested by contigs corresponding to acyltransferases, hydroxycinnamoyltransferase, hydroxycinnamoyl-CoA acyltransferase, deacetoxyvindoline acetyltransferase). This family of enzymes catalyzes acyl transfer reactions between CoA-activated hydroxyl-cinnamic acid derivatives and hydroxylated aliphatics [48]. The expression of BAHD acyltransferase encoding genes has been correlated with the biosynthesis of cutin and suberin [48]. The up-regulation of these genes accounts for the production of long chain fatty acids that are the precursors for cuticle formation [46].

*MYB* (*my*elo*b*lastosis), *MYB*-related, *MYB*-like coding genes were up-regulated in *P. purpurea* challenged with *P. cinnamomi.* MYB families have been shown to be important regulators of both cuticle biosynthesis and epidermal cell differentiation, highlighting the connection between these processes [49–52]. The role of a MYB transcription factor as regulator of VLCFAs (*v*ery *l*ong *c*hain *f*atty *a*cids) biosynthesis has also been demonstrated [53].

ABC (*A*TP-*B*inding *C*assette) transporters were also up-regulated. Recently, transporters required for cutin deposition were identified in *Arabidopsis* (ABCG32) [54] as well as in wild barley (*Hordeum spontaneum*) and rice (*Oryza sativa*) [55]. There is clear genetic evidence supporting a role for ABC transporters in cuticular lipid export [46]. An ABC transporter localized at the plasma membrane of epidermal cells of *Arabidopsis* suggests that ABCG32 exports cutin precursors for the synthesis of the cuticular layer in the epidermal cells [54].

The most common hemicellulose in the primary cell wall of plant epidermis is xyloglucan [56]. Genes involved in its integration in cell walls such as xyloglucan:xyloglucosyl transferase and in extension of isolated cell walls like α-expansin were up-regulated, suggesting also a response to reinforce the cell wall barrier against the penetration of *P. cinnamomi* [57]*.*

#### Casparian strips

*Phlomis purpurea* presents constitutive Casparian strips in the exodermal cells. Casparian strip polymer is made from monolignols and consists either of conventional lignin or of a very similar lignin-like structure. Casparian strips generate a para-cellular barrier, analogous to tight junctions in animals, that is thought to be crucial for selective nutrient uptake, exclusion of pathogens and many other processes [10, 13]. There is considerable debate regarding the chemical nature of Casparian strips, both suberin and lignin being considered as major components. Major current surveys describe the Casparian strips as an essentially suberin-based structure [58, 59]. However, in *Arabidopsis* Casparian strip diffusion barrier is made of a lignin polymer without suberin [10, 14]. Many, but not all plants have hypodermal/exodermal cell layers below the epidermis that are highly lignified and suberized and act similarly to the Casparian strips [13].

The molecular mechanisms regulating Casparian strips formation have been studied only in the endodermis. In this study we assume that the formation of exodermal Casparian strips may be regulated in a similar way as the endodermal Casparian strips. Selecting a few genes from the transcriptome assembly and combining them with histochemical observations of *P. purpurea* challenged and not-challenged with *P. cinnamomi* enabled us to correlate them with the up-regulation of lignin compounds in *P. purpurea* roots. A constitutive deposition is evident and genes involved in lignin biosynthesis appear up-regulated upon challenge. The cinnamoyl CoA:NADP oxidoreductase coding gene accounts for an important role in the first steps of lignin biosynthesis in *P. purpurea*. Cinnamoyl CoA:NADP oxidoreductase (CCR) catalyzes the conversion of cinnamoyl CoA esters to their corresponding cinnamaldehydes, i.e. the first specific step in the synthesis of the lignin monomers [60] whereas cinnamyl alcohol dehydrogenase catalyses the final step in the biosynthesis of monolignols [61]. Other enzymes of the lignin biosynthetic pathway, namely the cytochrome P450 enzymes, cinnamate 4-hydroxylase, 4-coumarate 3-hydroxylase, phenylalanine ammonia-lyase [62] appear also up-regulated.

In the present study, genes encoding for laccases were found up-regulated. In vascular plants, laccases are involved in lignification of xylem tissues [63]. Laccases play an essential role in developmentally regulated vascular lignification, namely in Casparian strips [14]. The study of the *Arabidopsis* laccase4 (lac4) and lac17 double knockout mutant provided the first in vivo evidence that these two laccase genes are involved in monolignol polymerization [64]. Laccase is necessary and non-redundant with peroxidase for lignin polymerization [65].

In what concerns peroxidases, it has been reported that peroxidase is required for monolignol oxidation in the Casparian strip [12]. The same authors have also pointed out that subcellular precision of lignin deposition in the endodermis is achieved by localized peroxidase expression and locally restricted production of reactive oxygen species [66].

*CASP*-like genes were also up-regulated in both control and challenged *P. purpurea*. These genes have been described by Roppolo et al. [66] and a function in endodermal Casparian strip formation has been assigned on the basis of a study using a combination of microarrays, immune-electron microscopy and *CASP* mutants in *Arabidopsis.* According to these authors they are probably involved in the formation of membrane protein platforms for the localization of cell-wall-modifying activities or localized adhesion [66].

*MYB* genes, discussed above as having a role in cuticle biosynthesis, are also transcriptional activators of the lignin biosynthetic pathway [67]. The transcription factor MYB36 has been shown to control the expression of the machinery required to polymerize lignin in the cell wall for the formation of the Casparian strip in *Arabidopsis* [68] and PtrMYB152, a poplar homolog of the *Arabidopsis* R2R3 MYB transcription factor, has been considered to play a role in the regulation of secondary cell wall biosynthesis [69].

The results under discussion account for the first correlation between constitutive morphological features and genes putatively involved in cutin biosynthesis and exodermal Casparian strips formation in *P. purpurea* control and challenged by *P. cinnamomi* which accounts for a constitutive resistance of *P. purpurea* to the root oomycete pathogen. The up-regulation of genes involved in these biosynthetic processes suggests that *P. purpurea*, in spite of having a constitutive defence mechanism against *P. cinnamomi*, is able to activate defence responses when challenged with this oomycete. Other authors have reported on the anatomical distribution of Casparian strips in different stages of endodermis formation as well as on the identification of soybean root suberin (preformed or pathogen induced) and its relationship to partial resistance to *Phytophthora sojae* [70, 71].

### Other genes related to defence response

Several genes involved in regulation of plant immune response or stress adaption were up-regulated in *Phlomis purpurea* after challenge with *Phytophthora cinnamomi*, showing that in addition to a resistance involving structural barriers, other signaling events induced by the pathogen, activate defence mechanisms in the plant. Some examples are described below and shown in Table 3.

It is the case of *AP2* (APETALA 2), a gene that belongs to a large multigene family that appear to act as key regulators in developmental processes and to be involved in responses to different biotic and abiotic stresses [72]. AP2/EREBP (AP2/ethylene response element binding protein) transcription factors have a function in stress signal integration and retrograde signaling and are linked to gene networks as determined from transcriptional profiling-based graphical Gaussian models [73].

GDSL (Gly-Asp-Ser-Leu) enzymes were also up-regulated. The GDSL esterases/lipases participate in many cellular processes such as plant development, morphogenesis and defence from pathogens and stress [74]. The involvement of gdsl genes in plants is still poorly understood. Recently it has been subject of much attention in studies regarding for example, gene family diversity or structural and functional studies [75–77].

Saponins are complex glycosylated triterpenes synthesized from mevalonate via farnesyl diphosphate and squalene [78, 79]. They are known to provide protection against pathogens and pests [80, 81]. Detergent-like properties of *P. purpurea* probably arise from the surfactant properties of saponins [78], suggesting that this plant is rich in these compounds and that they may participate in its defence mechanism against *P. cinnamomi*. Some unigenes (glycosyltransferases, UDP- glycosyltransferase, acyltransferases, farnesyl synthase, squalene synthase, cyclase, terpene, brassinosteroid, β-amyrin synthase) putatively related to enzymes involved in the biosynthetic routes to triterpenes or to their glycosylated form were up-regulated, suggesting a response from *P. purpurea* to activate their synthesis upon challenge with the pathogen,

Pathogen effectors are recognized by NB-LRR proteins that mediate disease resistance against pathogens which can grow only on living host tissue (obligate biotrophs), or hemibiotrophic pathogens [82, 83]. *P. cinnamomi* belongs to this last category and appears to deliver effectors that activate cc-nb-lrr genes in *P. purpurea.*

Production of pathogenesis related protein (PR-proteins), reinforcement of plant cell walls and the accumulation of phytoalexins are among defence responses induced pathogens leading to the activation of defence-related genes [84]. In *P. purpurea,* genes encoding at PR-proteins appear to be up-regulated at 12 h post-challenge with *P. cinnamomi*.

## Conclusions

This paper reports on the transcriptional response of *Phlomis purpurea* when challenged with the oomycete *Phytophthora cinnamomi.* The mRNA expression profiling of *P. purpurea* should further contribute for the understanding of essential features of *P. purpurea* biology, in particular transcriptional responses underlying pathogenic oomycete/plant interaction capable of being used in plant protection practices. The identification of marker or candidate genes such as those correlated with biotic stresses may be useful for field diagnostic, or for the understanding of defence mechanisms, including immunity and disease resistance in different plant species. The results here reported may also be useful in programs of environmental friendly soil disease control.

In short, the data obtained provide a good platform for additional studies on functional genomics of *P. purpurea* and for identifying candidate genes involved in defence against pathogens. This is of particular relevance since *P. purpurea* root cells are not penetrated by the highly aggressive pathogen and the plant is known to drastically reduce the growth of *P. cinnamomi* inoculum in the soil by producing metabolites that inhibit the formation of this soil-born pathogen disease cycle structures.

## Methods

### Seedling production

*Phlomis purpurea* seeds were collected in the field, across the Algarve, southern Portugal. They were stored at 4 °C until processed (from zero to 12 months as they were being used). Prior to germination, the seeds were surface sterilized with sodium hypochlorite 25% for 25 min and rinsed twice in sterile distilled water (SDW). The moistened seeds were covered with wet absorbent paper in Petri dishes until germination occurred (1 week). When the radicles were 2–3 cm long (24 to 48 h) they were transferred into cylindrical soft black plastic tubes (25 cm × 3 cm) containing vermiculite.

### Zoospore production

Zoospores were produced under aseptic conditions following a modification of the procedure reported by Byrt & Grant [85], as follows. Briefly, a 5 mm diameter *Phytophthora cinnamomi* culture plug was transferred onto 10% V8 juice agar medium (V8A) and incubated for 3 days, at 24 °C. Five small V8A plugs from the edge of the actively growing colony in Petri dishes were transferred to Miracloth membranes (Calbiochem-Novabiochem, Alexandria, Australia) overlying the agar medium. The cultures were incubated for 15 days at 24 °C. The Miracloth support and mycelia were transferred to 100 ml 5% V8 broth (V8B) and the culture shaken overnight (16 h) at 90 rpm at 24 °C. The nutrient medium was then replaced with a mineral salt solution (MSS) consisting of 0.01 M Ca(NO_3_)_2_.4 H_2_O, 0.005 M KNO_3_ and 0.004 M MgSO_4_.7H_2_O dissolved in 1 l of distilled water, autoclaved subsequently supplemented with 1 ml 0.1 M C_10_H_12_N_2_NaFeO_8_ solution, previously sterilized through a 0.22 μm filter (Millipore®). The culture was then shaken for 24 h. Sporangia were induced to release zoospores by incubating the Miracloth covered with MSS in Petri dishes at 4 °C for 20 min. Then, the Petri dishes were exposed to fluorescent light (Philips TLD 30 W/54) at room temperature, for 3 h. The zoospore suspension from each Miracloth was transferred into a 15 ml conical tube. The upper 2 ml were transferred to a second tube and shaken for 70 s to have zoospores encysted. Zoospore concentration was determined using a haemocytometer and the suspension diluted with SDW to 10^4^ zoospores ml^−1^ (Fuchs-Rosenthal). With this method, 10^4^–10^5^ zoospores ml^−1^ were routinely produced.

### Challenging with zoospores

Two and a half month old seedlings obtained from *P. purpurea* seeds were carefully removed from the vermiculite by immersion in water to wash out the vermiculite from the root system. The intact seedlings were placed in distilled water at 22 °C and challenged immediately by root-dip into 50 ml of a 10^4^ ml^−1^ zoospore suspension in MSS for 0, 6, 12, 24, 48 and 72 h. Controls were made with MSS at the same time points. Plants and controls were kept in glass tubes in the dark at 22 °C. At each time point, the whole plants were harvested and immediately frozen in liquid nitrogen and kept at −80 °C.

Three plantlets for each time point were pooled. Three replications of the whole experiment were carried out (three replicates).

### Tissue processing for microscopic studies

For the preparation of resin blocks, control and infested 2.5-month-old *P. purpurea* roots were cut in ca 5 mm long fragments at 0, 12, 24, 48 and 72 h post infestation (hpi) with *P. cinnamomi* mycelia or zoospores. *P. purpurea* control and infested roots were washed in distilled water and fixed with Karnovsky [5% glutharaldehyde +5% paraformaldehyde in phosphate buffered saline (PBS), pH 7.2] for 12 h at 4 °C [86]. The samples were washed 3 times with PBS for 10 min each. Samples were post-fixed with 2% osmium tetroxide (OsO_4_) in 0.02 M PBS, pH 7.2 for 2 h at room temperature in a glass container. After washing in buffer (3 times with PBS for 10 min each), samples were dehydrated in graded series of ethanol. Four seedlings were used for each condition. After, the samples were introduced into a mixture of propylene oxide:embedding agent (Spurr; ref. 14,300; Electron Microscopy Science, Hatfield, PA, USA) (2:1, v/v), stirred and allowed to stand for 2–3 h. The mixture was replaced with a 1:1 propylene oxide:embedding agent overnight and then replaced with a 1:3 propylene oxide:embedding agent for 2–3 h. A 100% Spurr was added and allowed to stand overnight. The samples with pure resin were placed in propylene flat embedding molds (EMS 70905) and oriented appropriately to be cut transversely or longitudinally. The molds were inserted into box cocoon BE8 (EMS 64300) and closed to prevent access of oxygen to the samples. Curing took 16–24 h at 60 °C. Semi-thin sections of 2 μm thick were made using an ultramicrotome (Ultratome Nova LKB Bromma) with a diamond knife DIATOME Histo 45°. The sections (16–20) were stained with 0.5% aqueous toluidine blue and observed under an Olympus Provis AX-70 light microscope (Olympus Corp., Japan). Images were obtained through an Infinity 2 CCD (Lumenera Corp., Ottawa, ON, Canada) digital camera and processed by Lumenera AnalySIS software.

For the preparation of paraffin blocks the root segments were fixed with FAA (5 ml formaldehyde 38%, 5 ml glacial acetic acid and 90 ml ethanol 70%) in 2.0 ml tubes with screw lids. After four roots of each sample (condition) were dehydrated by means of an ethanol series and finally treated twice with isoamyl acetate for 30 min, with xylene saturated with paraffin at 60 °C for 5 min and with pure paraffin for 20 min before embedding in vertical and horizontal position, in liquid paraffin (60 °C). Histological sections (12–14 μm) of paraffin embedded samples were performed using a microtome (Microm–HM 340 E). Then, the sections were deparaffinised, hydrated through graded ethanol series and stained with direct red (in 0.1–0.01 mg/ml PBS pH 7.2). After 20 min, staining was followed by brief washing in PBS. A total of 10 slides per sample were made. Observations were performed using an Olympus Provis AX-70 light field with an epifluorescence system Olympus U-ULS 100 HG. For epifluorescence observations, a cube U-MWBV (excitation filter: 400–440 nm, barrier filter: 475 nm) and a cube U-MWU (420 nm barrier filter, 330–385 nm excitation filter) were used. The images were taken in the same way that for semi-thin cuts.

In order to compare the thickness of the radial walls of the exodermis and the outer tangential walls between the control and challenged root samples, we used as statistical analysis a T test comparing a total of 50 control samples and 50 challenged samples.

For the observations with low temperature scanning electron microscopy (LTSEM) root samples were washed with abundant distilled water and frozen with liquid nitrogen. Then, samples were inserted into a cryo-observation system, for 15 min and coated with gold for 30 s. The samples were observed under a JEOL JSM microscope 5410, model LTSEM Service UPV microscopy. Images were captured with a digital camera Altra 20 and treated with imaging software GetIt analysis1.

### RNA extraction and cDNA libraries construction

Samples consisting of a pool of three whole plantlets per each challenging time point and per each one of three independent experiments (a total of 18 samples) were used independently to isolate total RNA. The total RNA was isolated using the RNeasy Mini Kit (Qiagen) following the manufacturer’s instructions. The concentration and quality of RNAs were determined using a NANODROP 2000C equipment (Thermo Scientific NanoDrop 2000C) and 1% agarose gel electrophoresis. After quantification, an equal amount of each biological RNA replicate was used to make 3 pools of each time point, corresponding to three independent experiments (repetitions). The total of 18 pools (10 μg of total RNA each) were used to construct *Phlomis purpurea* cDNA libraries that were sent to the Roslin Institute (University of Edinburgh) for paired-end mRNA sequencing with Illumina HiSeq™ 2000 platform.

### Sequencing and assembly

A 100 bp paired-end sequencing protocol with insert sizes from 165 to 182 bp, at the Roslin Institute, was used. Before introducing the reads into the assembly pipeline, the raw sequence reads were filtered and quality examined following steps: (1) removing the adapters using FASTXtoolkit pipeline version 0.0.13 [87]; (2) discarding the low quality reads to ensure that more than 70% bases of each retained read possess Illumina quality greater than 30 (q30 indicates 1% sequencing error rate) using NGS QC Toolkit version 2.2.2 [88]; (3) examining the quality of the reads using FastQC [89].

#### De novo *assembly*

For assembly, a de novo approach using Velvet Oases pipeline by setting Kmer length from 21 to 57 with a step length of 4 to re-predict transcripts. The data obtained for Kmer 39 were kept for further Velvet Oases [26, 27] analysis to maximize N50 and minimize the number of unused reads. The popular assemblers were used after merging the reads with Flash for de novo assembly of *P. purpurea* transcriptomic data. The Velvet program designed for DNA assembly has been widely used in many studies to explore the effect of different hash lengths (k-mer) on isoform detection [27, 90, 91]. To assemble short reads from RNA, the Oases programme was applied to increase the sensitivity of isoform detection.

The all-unigene transcripts obtained from the assembly were then used for subsequent downstream analysis. The assembled unigenes, with sequence length longer than 200 bp, was deposited in the NCBI Sequence Read Archive (SRA) under the accession number SRP046996.

### Functional characterization and gene ontology (GO) annotation

The sequence orientations of the unigenes were determined by BLASTX against the NCBI Nr and the Swiss-Prot protein databases, the Kyoto Encyclopedia of Genes and Genomes (KEGG) pathway and the Cluster of Orthologous Groups (COG) databases. The incongruent results from different databases were settled under the priority order as listed in the precedent sentence. For the remaining unigenes that were unaligned to the above databases, ESTScan was used to predict their sequence orientations. For assignments of gene descriptions, the unigenes were searched against the Nr database using BLASTX with an *E-*value cut-off of 1xe^−5^. Only results with the best hit were selected.

### Functional classification by KEGG

Based on Nr annotations, the genes of non-plant origin were removed. The remaining plant genes were kept for further analysis. The unigenes were assigned GO annotations using Blast2GO. The putative metabolic pathways for the unigenes were assigned by performing BLASTX against the KEGG pathway database with the *E*-value cut-off of 1xe^−5^. All the mapped sequences were annotated to the KEGG database to obtain the enzyme commission (EC) number. The ECs were then mapped to the KEGG Pathway to obtain the KEGG Pathway-Maps followed by functional classification using the Blast2GO software [92].

### GC content analysis

The GC content analysis provides insights into various aspects related to organism genome, including evolution, gene structure (intron size and number), thermostability and gene regulation [93–95]. GC content analysis was done using the PRINSEQ (PReprocessing and INformation of SEQuences) [96] tool to generate statistics of sequence data for sequence length, GC content and sequence with Ns.

### Differential expressed genes analysis using DESeq

The comparison of differential gene expression profiles was conducted using the DESeq package from Bioconductor [97] and the 18 cDNA libraries from *P. purpurea* plants challenged with *P. cinnamomi* at six post-challenge time points (0, 6, 12, 24, 48 and 72 h). It was done normalizing *tag* distribution for gene expression level in each library to make an effective library size and extract significance of differentially expressed transcripts (DETs) with *p*-value 0.05 and log2 fold-change. DESeq provides an empirical approach and eliminates introduced bias resulting from RNA composition, according to the user manual.

The DESeq package in R was applied to data replicated in our local Galaxy server. Briefly, we started by acquiring reads through a Quality Assurance and Improvement pipeline and imported the transcript file (all treated pairs and all untreated pairs of reads) into Galaxy server history. The libraries were set to *fastqsanger* using *fastq* Grooming and then mapped to the annotated *P. purpurea* transcriptome using BWA [98]. After finishing the BWA mappings the counts table was created using SAM to Count package from NGS and then the DESeq was run. The counts table produced from the SAMTools to Count Package was used to produce a top-table file and diagnostic plots which include “Dispersion Estimates”, “Base Mean vs. Log2 Fold Change”, “*P*-value histogram”, “Top 100 Genes/Transcripts by *P*-value”, “Moderated LFC vs. LFC”, “VST Sample Clustering” and “MDS Plot”.

### Quantitative real-time PCR analysis

qRT-PCR analysis of 8 differential expressed genes was performed using beta-actin, succinate dehydrogenase and elongation factor1 alpha from *P. purpurea* as reference genes. Before selecting the control genes we checked that expression of these genes was not affected by the challenging *P. purpurea* plants with *P. cinnamomi* at any time point. The selected genes are putatively related to *P. purpurea* response to the pathogen and include: wall-associated receptor kinase 5-like, cinnamyl alcohol dehydrogenase, hydroxyproline-rich glycoprotein, cyanidin-3-O-glucoside 2-O-glucuronosyltransferase-like, resistance protein rgc2, subtilisin-like protease, calcium-binding mitochondrial carrier protein aralar1-like and serine-threonine protein plant. Gene expression levels were compared separately between plants challenged with *P. cinnamomi* and non-challenged plants (controls) at three (12 h, 24 h and 48 h) post-challenge time points. *Phlomis purpurea* beta-actin, succinate dehydrogenase and elongation factor1 alpha were used as reference genes.

The total RNA (1 μg) was reverse-transcribed to cDNA using Maxima RT reagent Kit (Thermo Scientific) in a total volume of 10 μl, according to the manufacturer’s instruction. The gene-specific primers were designed based on the sequencing results and using the Primer Premier software (version 6.0) (Additional file 15: Table S9). Then, 20 μl of PCR samples containing 1 μl of first-strand cDNAs and 1 μl of forward and reverse primers were subjected to 35 cycles of 30 s denaturation at 94 °C, 40 s annealing at 58 °C and 30 s extending at 72 °C. The PCR products were electrophoresed on a 1% agarose gel.

The real-time PCR was performed on a StepOne Plus System (Applied Biosystems) using 4 μl of first-strand cDNAs diluted 1:40 and SYBR® Premix Ex Taq™ (TAKARA). The thermal cycling conditions were 30 s at 95 °C, followed by 40 cycles of 5 s at 95 °C and 15 s at 58 °C. All reactions were performed in quadruplicate. The relative expression cycles for each gene were calculated using the 2^-ΔΔCT^ methods with normalization to the internal control gene. The LSD-t test of one-way analysis of variance was performed to determine the significant differences in expression levels between the treated samples at 12 h, 24 h and 48 h and those at 0 h using the SPSS software package (version 13.0).

## Additional files

Additional file 1: Statistics of histological measurements. (DOCX 15 kb)

Additional file 2: Figure S1. Frequency distribution of lengths of transcript contigs resulting from Illumina HiSeq™ 2000 sequencing. (DOCX 61 kb)

Additional file 3: Figure S2. Homology of annotated *Phlomis purpurea* unigenes to genes from plants. (DOCX 144 kb)

Additional file 4: Figure S3. Gene ontology assignments for *Phlomis purpurea* transcripts. Distribution of *Phlomis purpurea* contigs into functional sub-categories of Gene Ontology (GO). (DOCX 4917 kb)

Additional file 5: Figure S4. GC content analysis of *Phlomis purpurea* transcripts. The average of GC content for *P. purpurea* was calculated to evaluate the percentage of transcripts with GC content within a range. (DOCX 54 kb)

Additional file 6: Table S1. GC content distribution of *Phlomis purpurea* transcripts. (DOCX 13 kb)

Additional file 7: Table S2. List of *Phlomis purpurea* unigenes up-regulated (fold increase > 2) at 24 h post-challenge with *Phytophthora cinnamomi*. (XLSX 557 kb)

Additional file 8: Table S3. List of *Phlomis purpurea* unigenes up-regulated (fold increase > 2) at 12 h post-challenge with *Phytophthora cinnamomi*. (XLSX 880 kb)

Additional file 9: Table S4. List of *Phlomis purpurea* unigenes up-regulated with Padj <0.05 at 24 h post-challenge with *Phytophthora cinnamomi*. (XLSX 98 kb)

Additional file 10: Table S5. List of *Phlomis purpurea* unigenes up-regulated with Padj <0.05 at 12 h post-challenge with *Phytophthora cinnamomi*. (XLSX 217 kb)

Additional file 11: Table S6. List of *Phlomis purpurea* unigenes up-regulated (fold increase > 4) at 24 h post-challenge with *Phytophthora cinnamomi*. (XLSX 162 kb)

Additional file 12: Table S7. List of *Phlomis purpurea* unigenes up-regulated (fold increase > 4) at 12 h post-challenge with *Phytophthora cinnamomi*. (XLSX 281 kb)

Additional file 13: Table S8. List of *Phlomis purpurea* unigenes up-regulated (fold increase > 10) at 24 h post-challenge with *Phytophthora cinnamomi*. (XLSX 58 kb)

Additional file 14: Table S9. List of *Phlomis purpurea* unigenes up-regulated (fold increase > 10) at 12 h post-challenge with *Phytophthora cinnamomi*. (XLSX 68 kb)

Additional file 15: Table S10. Specific primers used in qRT-PCR analysis of eight differential expressed genes from *Phlomis purpurea*. (XLSX 12 kb)

## Abbreviations

ABC transporters: ATP-binding cassette transporters
AP2: APETALA 2
BAHD: Benzylalcohol acetyltransferase, anthocyanin O-hydroxycinnamoyltransferase anthocyanin O-hydroxycinnamoyltransferase, anthranilate N-Hydroxycinnamoyl/benzoyltransferase, Deacetylvindoline acetyltransferase
BLAST: Basic Local Alignment Search Tool
bp: Base pair
CASP: Casparian
CCR: Cinnamoyl CoA:NADP oxidoreductase
cDNA: Complementary DNA
COG: Cluster of Orthologous Groups
CYPs: Cytochrome Enzymes
EC: Enzyme Commission number
EREBP: Ethylene response element binding protein
GDSL: Gly-Asp-Ser-Leu
GO: Gene Onthology
GPAT: Glycerol-3-phosphate acyltransferase
hpi: Hours post infestation
KEGG: Kyoto Encyclopedia of Genes and Genomes
LTSEM: Low Temperature Scanning Electron Microscopy
mRNA: Messenger RNA
MSS: Mineral salt solution
MYB: Myeloblastosis
NB-LRR: Nucleotide binding-leucine rich repeat
NCBI: National Center for Biotechnology Information
PBS: Phosphate buffered saline
PCR: Poly chain reaction
PRINSEQ: PReprocessing and INformation of SEQuences
PR-proteins: Pathogenesis related protein
qRT-PCR: Quantitative Real Time PCR
RNA: Ribonucleic acid
RNA-Seq: RNA sequencing technology
SDW: Sterile distilled water
SRA: Sequence Read Archive
UV: Ultra Violet
VLCFAs: Very long chain fatty acids
