# Supplementary material for: De novo assembly of Phlomis purpurea after challenging with Phytophthora cinnamomi
Source: BMC Genomics. 2017 Sep 6;18:700. doi: 10.1186/s12864-017-4042-6 (PMC5585901; doi:10.1186/s12864-017-4042-6)
Supplement: Supplementary file 1 — Statistics of histological measurements. (DOCX 15 kb) [file 12864_2017_4042_MOESM1_ESM.docx]

**Statistics of histological measurements**

**Thickness variation in radial walls exodermis (in microns)**

|  | CONTROL | CHALLENGED |
| --- | --- | --- |
| Mean | 0.47200 μm | 0.50300 μm |
| SD | 0.06900 | 0.11000 |
| SEM | 0.00976 | 0.01556 |
| N | 50 | 50 |

P value: 0.0946 (not statistically significant)

Means difference: -0.031

95% confidence interval of this difference: from -0.06744 to 0.00544

*t*: 1.6881

Df: 98

Standard error of difference: 0.018

**Thickness variation in external tangential walls of epidermis (in microns)**

|  | CONTROL | CHALLENGED |
| --- | --- | --- |
| Mean | 1.42100 μm | 2.92500 μm |
| SD | 0.40700 | 0.31500 |
| SEM | 0.05756 | 0.04455 |
| N | 50 | 50 |

P value: <0.0001 (statistically significant)

Means difference: -1.50400

95% confidence interval of this difference: from -1.64844 to 1.35956

*t*: 20.6639

Df: 98

Standard error of difference: 0.073
