# Supplementary material for: De novo assembly of Phlomis purpurea after challenging with Phytophthora cinnamomi
Source: BMC Genomics. 2017 Sep 6;18:700. doi: 10.1186/s12864-017-4042-6 (PMC5585901; doi:10.1186/s12864-017-4042-6)
Supplement: Supplementary file 2 — Frequency distribution of lengths of transcript contigs resulting from Illumina HiSeq™ 2000 sequencing. (DOCX 61 kb) [file 12864_2017_4042_MOESM2_ESM.docx]

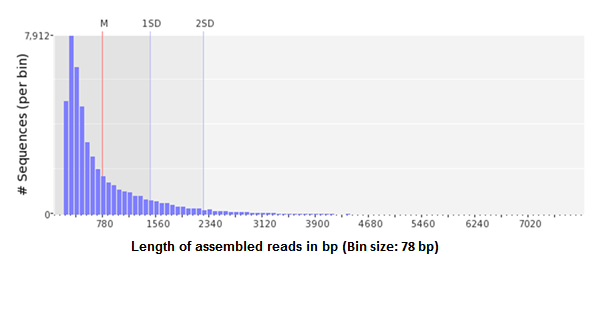


Figure S1 Frequency distribution of lengths of transcript contigs resulting from Illumina HiSeq™ 2000 sequencing.
