# Supplementary figures and images for: De novo assembly of Phlomis purpurea after challenging with Phytophthora cinnamomi
Source: BMC Genomics. 2017 Sep 6;18:700. doi: 10.1186/s12864-017-4042-6 (PMC5585901; doi:10.1186/s12864-017-4042-6)

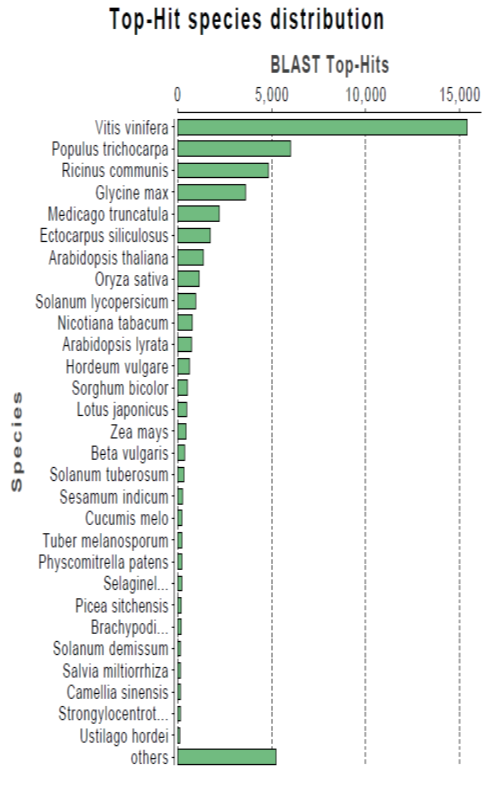


Figure S2. Homology of annotated *Phlomis purpurea* unigenes to genes from plants.

Supplement: Supplementary file 3 — Homology of annotated Phlomis purpurea unigenes to genes from plants. (DOCX 144 kb) [file 12864_2017_4042_MOESM3_ESM.docx]
