# Supplementary material for: De novo assembly of Phlomis purpurea after challenging with Phytophthora cinnamomi
Source: BMC Genomics. 2017 Sep 6;18:700. doi: 10.1186/s12864-017-4042-6 (PMC5585901; doi:10.1186/s12864-017-4042-6)
Supplement: Supplementary file 4 — Gene ontology assignments for Phlomis purpurea transcripts. Distribution of Phlomis purpurea contigs into functional sub-categories of Gene Ontology (GO). (DOCX 4917 kb) [file 12864_2017_4042_MOESM4_ESM.docx]

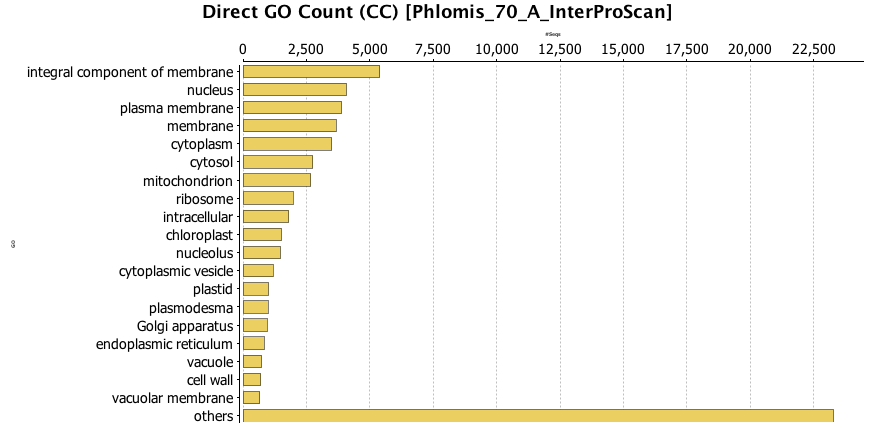

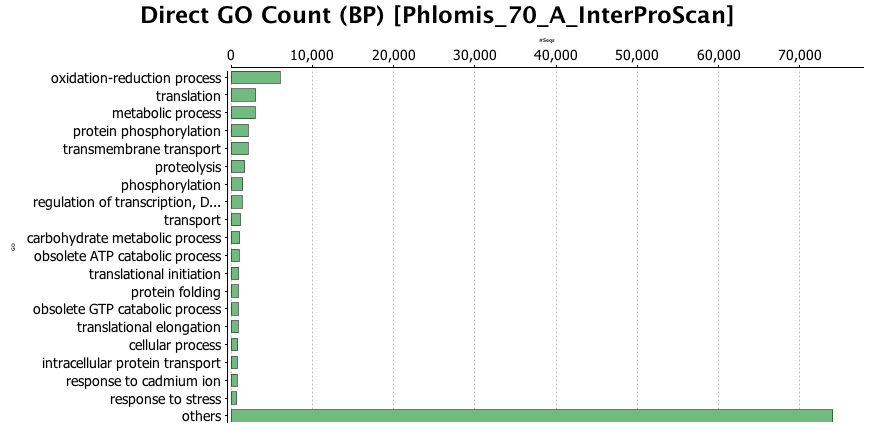

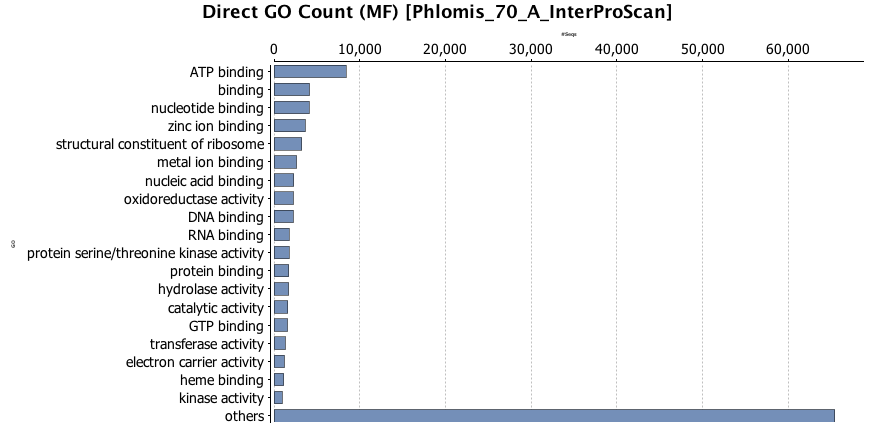


Cellular

Component

Biological

Process

Molecular

Function

Figure S3. Gene ontology assignments for *Phlomis purpurea* transcripts.

Distribution of *Phlomis purpurea* contigs into functional sub-categories of Gene Ontology (GO).
