# Supplementary material for: De novo assembly of Phlomis purpurea after challenging with Phytophthora cinnamomi
Source: BMC Genomics. 2017 Sep 6;18:700. doi: 10.1186/s12864-017-4042-6 (PMC5585901; doi:10.1186/s12864-017-4042-6)
Supplement: Supplementary file 5 — GC content analysis of Phlomis purpurea transcripts. The average of GC content for P. purpurea was calculated to evaluate the percentage of transcripts with GC content within a range. (DOCX 54 kb) [file 12864_2017_4042_MOESM5_ESM.docx]

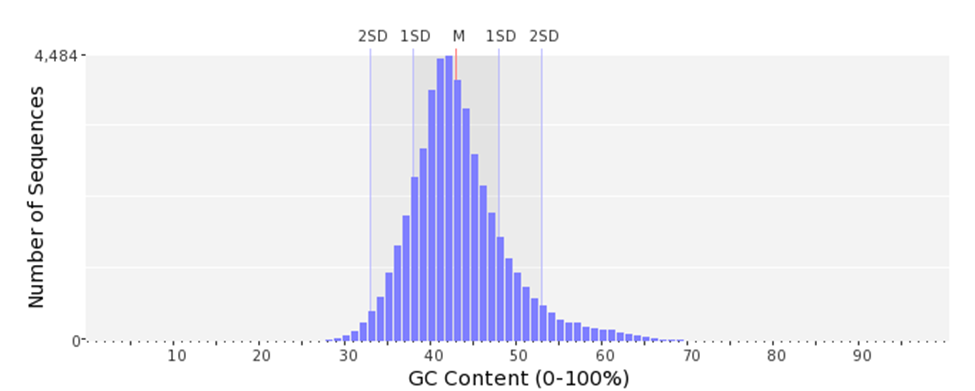


Figure S4. GC content analysis of *Phlomis purpurea* transcripts.

The average of GC content for *P. purpurea* was calculated to evaluate the percentage of transcripts with GC content within a range.
