# Supplementary material for: De novo assembly of Phlomis purpurea after challenging with Phytophthora cinnamomi
Source: BMC Genomics. 2017 Sep 6;18:700. doi: 10.1186/s12864-017-4042-6 (PMC5585901; doi:10.1186/s12864-017-4042-6)
Supplement: Supplementary file 6 — GC content distribution of Phlomis purpurea transcripts. (DOCX 13 kb) [file 12864_2017_4042_MOESM6_ESM.docx]

Table S1. GC content distribution of *Phlomis purpurea* transcripts.

**GC Content Distribution**

| Mean GC content: | **43.18 ± 5.74%** |
| --- | --- |
| Minimum GC content: | **26%** |
| Maximum GC content: | **73%** |
| GC content range: | **48%** |
| Mode GC content: | **42% with 4,483 sequences** |
|  |  |
